# Achieving a Near-Infrared Absorption by A−DA’D−A Type Isoindigo-Based Small Molecular Acceptors for Organic Photovoltaics

**DOI:** 10.3390/molecules30020344

**Published:** 2025-01-16

**Authors:** Hui Liu, Yu Wu, Dong-Nai Ye, Na Chen, Xu-Min Huang, Shi-Yong Liu

**Affiliations:** 1 School of Intelligent Manufacturing and Materials Engineering, Gannan University of Science and Technology, Ganzhou 341000, China; 9320230035@gnust.edu.cn; 2 School of Metallurgical and Chemical Engineering, Jiangxi University of Science and Technology, Ganzhou 341000, China; 18011263004@163.com (Y.W.); chenna0000@foxmail.com (N.C.); fengzhi70618@163.com (X.-M.H.); 3 School of Chemistry and Chemical Engineering, Gannan Normal University, Ganzhou 341000, China; yedongnai2014@gnnu.edu.cn

**Keywords:** isoindigo, near-infrared absorption, direct C–H arylation, acceptors, organic solar cell

## Abstract

Isoindigo (IID)-based non-fullerene acceptors, known for their broad absorption spectra and high charge carrier mobilities, play a crucial role in organic photovoltaics. In this study, two A−DA’D−A type unfused ring acceptors (URAs), IDC8CP-IC and IDC6CP-IC, were designed and synthesized using cyclopentadithiophene (CPDT) and IID core units, each functionalized with different alkyl chains (2-hexyldecyl and 2-octyldodecyl), through an atom- and step-efficient direct C–H arylation (DACH) method. Both URAs, despite the absence of non-covalent conformation locking between CPDT and IID, demonstrated favorable molecular planarity, broad absorption ranges, low band gaps, and high molar absorption coefficients. Notably, IDC6CP-IC exhibited stronger intermolecular charge transfer and J-aggregation. An organic solar cell (OSC) device based on IDC6CP-IC achieved a power conversion efficiency (PCE) of 3.10%, with a broad photoresponse range extending from 400 to 900 nm. This study highlights the significant impact of alkyl chain engineering on material synthesis, photoelectric properties, and corresponding device performance. Furthermore, DACH is shown to be a promising approach for synthesizing IID-based URAs with near-infrared (NIR) absorption, making it an excellent candidate for bulk heterojunction (BHJ) OSC applications.

## 1. Introduction

Organic solar cells (OSCs) have garnered significant attention as a renewable energy technology due to their advantageous properties, including their light weight, solution processability, excellent flexibility, and semi-transparency [1,2,3,4,5,6]. In recent years, non-fullerene acceptors (NFAs) have emerged as a key focus in the development of OSCs, owing to their distinct advantages such as broad absorption spectra, tunable energy levels, and ordered molecular packing [7,8,9]. The most prominent fused ring acceptors (FRAs), such as ITIC and Y6, feature multiple fused ring frameworks in their central cores, enabling power conversion efficiencies (PCEs) in single-junction OSCs to exceed 19% [10,11]. However, most FRAs face challenges related to complex and costly synthesis processes due to the need for constructing rigid coplanar core structures, which typically involve multiple fused aromatic rings with lateral side chains [12]. In contrast, unfused ring acceptors (URAs), where each unit of the central core is linked by a single bond, offer a more cost-effective and simpler synthetic route, making them attractive to researchers [13,14,15,16,17,18]. To date, single-junction OSCs based on URAs have achieved PCEs of up to 15.2% [19]. Notably, the formation of noncovalent intramolecular interactions, such as S···O, F···H, and F···S, in URAs with two or more aromatic rings can effectively constrain molecular twisting, resulting in more planar unfused aromatic rings and enhanced photovoltaic properties [17,20,21,22,23,24]. However, increased planarity in acceptors can lead to excessive aggregation, which may induce unfavorable phase separation. Therefore, it is crucial to pursue rational molecular design and reduce synthetic costs to balance planarity and rigidity in unfused conjugated structures for optimal performance.

**Figure 1:**
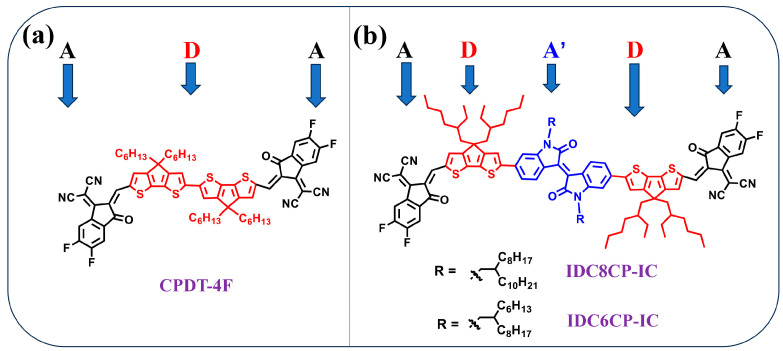
Chemical structures of (**a**) A−D−A type UFRA CPDT-4F and (**b**) A−D−A’−D−A type UFRAs IDC8CP-IC and IDC6CP-IC.

Most developed unfused ring acceptors (URAs), particularly A−D−A and A−DA’D−A type small molecules, have achieved excellent photovoltaic performance. Compared to A−D−A type URAs, A−DA’D−A type URAs introduce A’ units, creating multiple donor-acceptor (D−A) push–pull interactions. This allows greater flexibility in broadening the absorption spectra and adjusting the energy levels of URAs (Figure 1). The incorporation of the cyclopentadithiophene (CPDT) unit, which has a rigid planar structure, strong electron-donating capacity, and a high highest occupied molecular orbital (HOMO) energy level, acts as the “D” moiety connecting two adjacent acceptor groups. This design mimics the structure of ITIC. “A′” units, such as para-difluorobenzene [18], benzobis(thiazole) [25], and para-dialkoxybenzene [23], have been widely studied. A new series of A−DA’D−A type URAs, DF-PCIC, was first reported by Chen et al. in 2018, achieving an impressive power conversion efficiency (PCE) of 10.14% when combined with the polymer donor PBDB-T [18]. In 2021, Huang et al. developed BTzO-4F as the acceptor, which, when paired with the polymer PBDB-T, reached a PCE of 13.8% [26]. Isoindigo (IID), a strong electron acceptor with a deep lowest unoccupied molecular orbital (LUMO) energy level, is an amide-based unit used in OSCs [27]. IID shares advantages with pyrrolo-pyrrolidinedione (DPP), including good planarity, ease of synthesis, a deep HOMO energy level, and a strong electron-withdrawing ability [28]. IID is also readily available from natural sources and allows functionalization at multiple positions on its building blocks. The application of IID compounds in OSCs was first reported by Reynolds et al. in 2010 [29]. While most current research on OSCs focuses on IID-based polymers, small molecules based on IID for OSCs have been rarely reported, suggesting that IID can be introduced as an “A” unit in URAs [30,31,32]. On the other hand, fused ring acceptors (FRAs) achieve outstanding device performance due to their coplanar backbones, broad absorption ranges, and enhanced π−electron delocalization. This improves π−π stacking, charge transport, and enables absorption in the near-infrared (NIR) region. NIR absorption in acceptors plays a crucial role in enhancing OSC performance [33]. In general, the structural tunability of acceptors can be employed to adjust energy levels and absorption properties to create NIR absorbers [34]. However, achieving NIR absorption in URAs remains challenging. Therefore, designing A−DA’D−A type URAs with NIR absorption for high-performance OSCs is a significant challenge that requires further exploration.

It is established that the engineering of side chains in NFAs influences their processing capabilities and device fabrication due to variations in molecular packing and film morphology. These modifications can result in broad absorption, enhanced crystallinity, and consequently, improved device performance. In this study, two URAs, IDC6CP-IC and IDC8CP-IC, were synthesized. These URAs have IID and CPDT as the electron-accepting and electron-donating cores, respectively, and DFIC as the terminal groups. The synthesis involved direct C–H arylation (DACH) [35,36,37,38,39,40], the Vilsmeier–Haack (V–H) reaction, and a Knoevenagel condensation. This work explored the impact of different alkyl chains (2-hexyldecyl and 2-octyldodecyl) on IID for molecular aggregation and stacking. Both URAs demonstrated broad absorption extending into the near-infrared (NIR) wavebands. IDC6CP-IC exhibited the highest exciton dissociation efficiency, the most balanced charge transport, and achieved the highest power conversion efficiency (PCE) of 3.10%. The findings indicate that light absorption, frontier orbital energy levels, and molecular stacking can be modulated by controlling the alkyl chain length on the IID, thereby optimizing device performance. Additionally, the presence of NIR wavebands in IDC6CP-IC and IDC8CP-IC may enhance photon absorption capabilities and offer significant value in the development of semi-transparent organic solar cell devices. In this study, IID-based URAs based on different alkyl chains were developed, which have a significant impact on material synthesis, optoelectronic properties, and the performance of corresponding devices. Moreover, atom- and step-economic DACH was expected to be a promising synthetic method for URAs with near-infrared (NIR) absorption.

## 2. Results and Discussion

### 2.1. Synthesis and Characterization

**Scheme 1:**
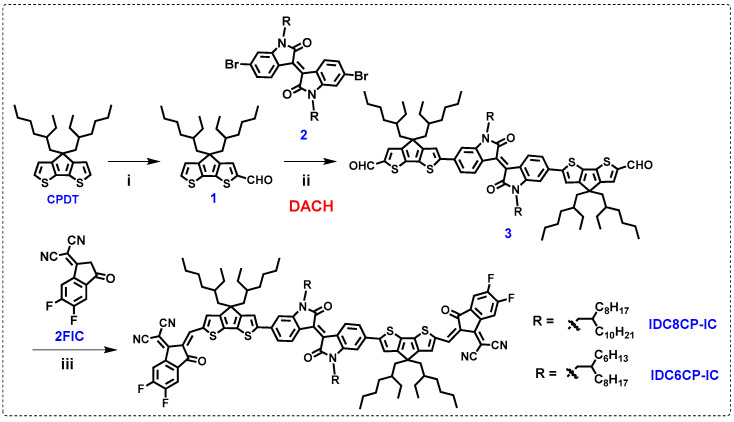
Synthetic route of IDC8CP-IC and IDC6CP-IC.

**Figure 2:**
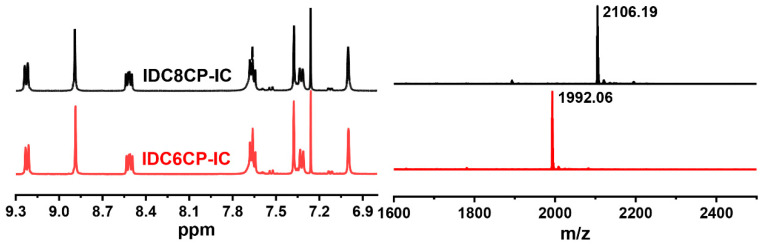
^1^H NMR spectra (**left part**) in the range of 6.9–9.3 ppm, and molecular ion peaks of MALDI-MS spectra (**right part**) of IDC8CP-IC and IDC6CP-IC.

Both URAs, IDC8CP-IC and IDC6CP-IC, exhibit good solubility in common organic solvents and were synthesized using identical methods. These compounds were prepared through direct C–H arylation, where brominated IID [34] was reacted with monoaldehyde CPDT [18] in a ratio of 1:2.2 to form the reaction precursors. These intermediates were then subjected to a Knoevenagel condensation reaction to yield the final products (Scheme 1). The chemical structures of both acceptors were analyzed using nuclear magnetic resonance spectroscopy (^1^H NMR) and high-resolution mass spectrometry (MS) (Figure 2 and Figures S2–S7). Both URAs displayed similar chemical shifts in ^1^H NMR, primarily due to their identical conjugated backbones, indicating that the length of the alkyl chains does not significantly affect the chemical shifts. As illustrated in Figure 2, mass spectrometry confirmed that the single molecular ion peaks of IDC8CP-IC and IDC6CP-IC correspond to their calculated molecular weights (MW) of 2106.19 and 1992.06, respectively.

**Figure 3:**
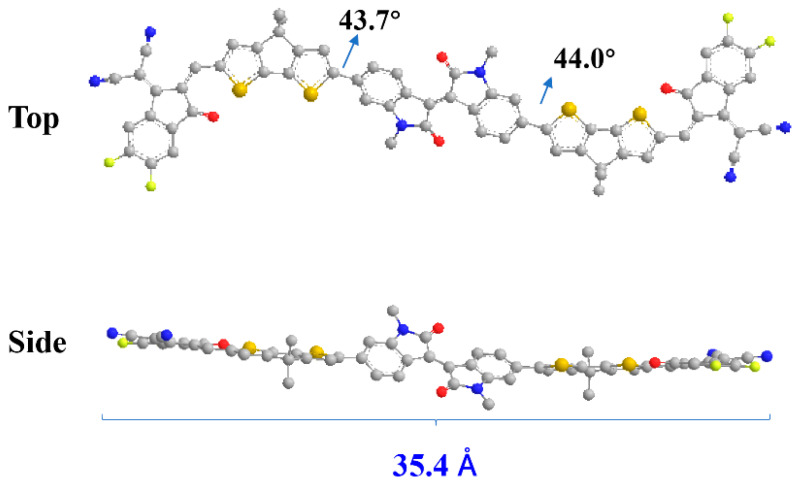
Top and side views of molecular geometries and optimized geometries calculated by density functional theory (alkyl substituents on the CPDT and IID units of the acceptors are replaced by methyl groups for simplifying the calculation) for IDC8CP-IC and IDC6CP-IC.

Density functional theory (DFT) calculations (B3LYP/6−31G*) were employed to elucidate the structure–property relationships of both URAs (Figure 3). Theoretical calculations based on DFT methods have been performed for the compounds with a Gaussian 09 program. Becke’s three-parameter gradient corrected functional (B3LYP) with the 6−31G (d,p) basis set was used for geometric optimization [41,42]. Prior to the theoretical calculations, the molecular structures were optimized by substituting all alkyl chains with methyl groups to facilitate the analysis. The DFT results show that the backbone lengths of both URAs measure 35.4 Å. The minimal dihedral angle between IID and CPDT suggests a well-planarized backbone, which enhances charge transport capabilities. From the side view of the molecular structure, it is observed that CPDT aligns more effectively with the diisocyanoalkane (DFIC) end groups. This alignment is primarily due to the noncovalent S···O interactions, which stabilize the planar conformation and contribute to the improved conjugation in both IDC8CP-IC and IDC6CP-IC.

### 2.2. Optical and Electrochemical Properties

**Figure 4:**
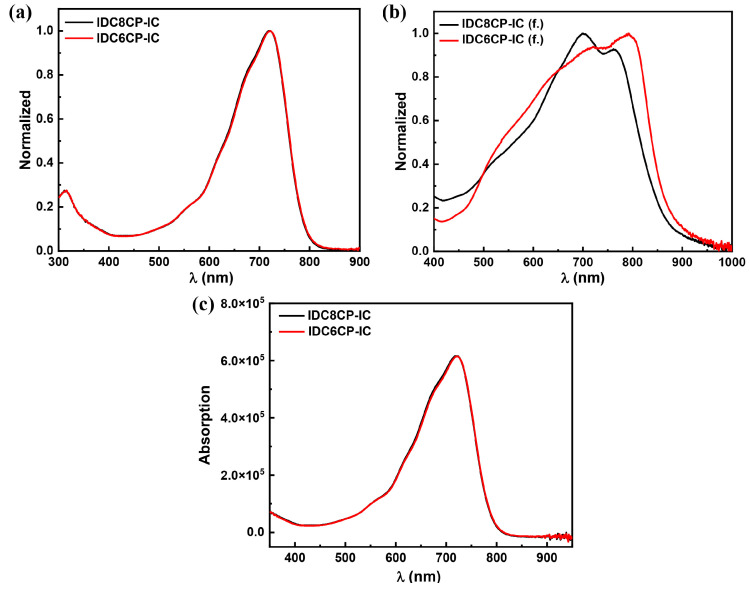
(**a**) UV–vis spectra of IDC8CP-IC and IDC6CP-IC in CH_2_Cl_2_ solution, (**b**) in solid film, and (**c**) in CH_2_Cl_2_ solution in 10^−6^ M.

The optical properties of both URAs, IDC8CP-IC and IDC6CP-IC, were investigated using ultraviolet-visible spectroscopy (UV-vis) (Figure 4), and the results are presented in Table 1. In solution, both URAs display nearly identical absorption peaks at approximately 721 nm, with high molar absorption coefficients of 6.16 × 10^5^ for IDC8CP-IC and 6.15 × 10^5^ M^−1^ cm^−1^ for IDC6CP-IC (Table 1 and Figure 4c), indicating strong intramolecular charge transfer capabilities. As depicted in Figure 4b, the absorption spectra of both URAs in film form exhibit significant red shifts and broader absorptions compared to their solution states, attributable to enhanced π−π stacking interactions within the solid films. Specifically, IDC8CP-IC and IDC6CP-IC show maximum absorption peaks at 700.0/761.5 nm and 720.0/789.5 nm, respectively. Notably, IDC6CP-IC demonstrates stronger and more pronounced absorption shoulder peaks, indicative of more robust intermolecular charge transfer, J-aggregation, and higher crystallinity. Additionally, IDC6CP-IC exhibits minor peaks at shorter wavelengths, primarily due to the modulation of intramolecular charge transfer by the IID nucleus, which slightly inhibits transitions between the HOMO and the LUMO. This results in coexistent HOMO-LUMO transitions that facilitate broad absorption across both long and short wavelengths. These features enable both URAs to complement the maximum absorption wavelength of the donor polymer PBDB-T, enhancing photon capture. Among them, IDC6CP-IC, with the most red-shifted and distinct shoulder peak, also absorbs in the 400–500 nm range, suggesting superior photon absorption capabilities. The optical band gaps (E_g_^opt^) of IDC8CP-IC and IDC6CP-IC were determined to be 1.38 eV and 1.33 eV, respectively, with IDC6CP-IC exhibiting the narrowest band gap and thus higher photon absorption efficiency.

**Figure 5:**
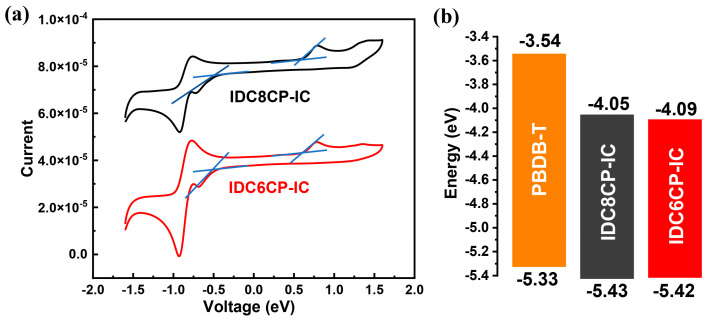
(**a**) CV curves of IDC8CP-IC and IDC6CP-IC and (**b**) FMOs level diagram of IDC8CP-IC, IDC6CP-IC, and PBDB-T.

Cyclic voltammetry (CV) was employed to evaluate the electrochemical properties of IDC8CP-IC and IDC6CP-IC and to estimate their frontier orbital energy levels. The cyclic voltammetry curves for both URAs are depicted in Figure 5. The energy level of the Ag/AgCl electrode was determined to be −4.61 eV, derived from the vacuum energy level of the ferrocene redox potential. The lowest unoccupied molecular orbital (LUMO) levels were estimated from the initial reduction potentials (*E*_red_) of CV plots, which were calibrated by the ferrocene-ferrocenium (Fc/Fc^+^) redox couple and calculated from the equation: *E*_LUMO_ = −4.80 − (*E*_red_ − *E*_Fc_/_Fc_^+^), where *E*_Fc_/_Fc_^+^ is the redox couple of Fc/Fc^+^ (here is 0.19 V, Figure S8). The highest occupied molecular orbital (HOMO) used a similar method and was calculated. The LUMOs/HOMOs of IDC8CP-IC and IDC6CP-IC obtained are −4.05/−5.43 and −4.09/−5.42 eV, respectively (Figure 5 and Table 1). Figure 5b illustrates the energy level distributions for IDC8CP-IC, IDC6CP-IC, and PBDB-T. Both URAs exhibit very similar energy levels, with IDC6CP-IC displaying a slightly higher HOMO and a deeper LUMO compared to IDC8CP-IC.

### 2.3. Photovoltaic Performances and Mechanism Study

**Figure 6:**
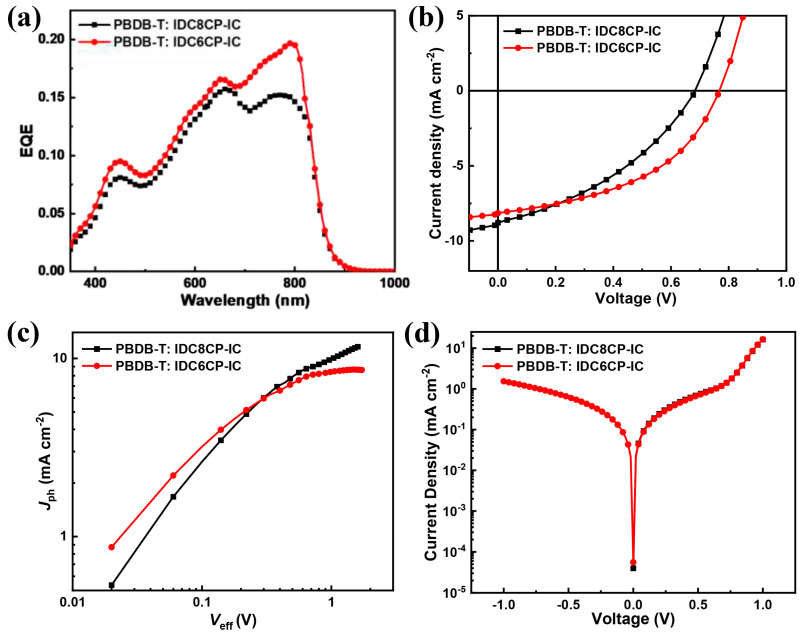

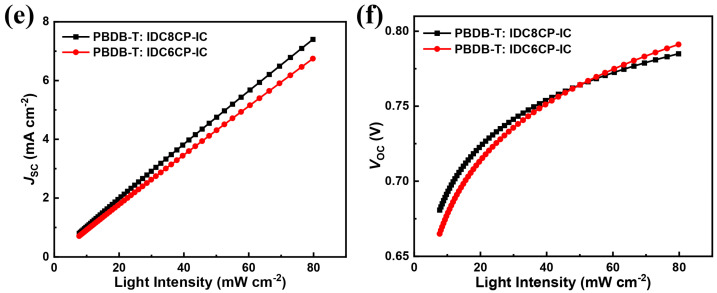
(**a**) EQE curves of PBDB-T: IDC8CP-IC and PBDB-T: IDC6CP-IC based BHJs; (**b**) *J−V* curves; (**c**) photocurrent density (*J*_ph_) dependence of V_eff_; (**d**) dark current of PBDB-T, IDC8CP-IC and IDC6CP-IC their BHJs; and light intensity dependence of (**e**) *J*_SC_ and (**f**) *V*_OC_.

To further examine the impact of the alkyl chain length in IID on the photovoltaic performance of both URAs, OSCs were fabricated using the structure ITO/PEDOT:PSS/PBDB-T: URAs/PDINO/Ag. IDC8CP-IC and IDC6CP-IC were employed as electron acceptors, PBDB-T as the donor material, and 1-chloronaphthalene (1-CN) as the additive. Optimal photovoltaic performance was achieved after adjusting the donor-acceptor ratio, donor solution concentration, annealing temperature, spin-coating speed, and additive content. The optimal conditions included thermal annealing at 100 °C. External quantum efficiency (EQE) spectra and *J−V* curves of the devices are depicted in Figure 6a,b, and the device parameters are summarized in Table 2. For devices utilizing IDC8CP-IC, featuring a longer alkyl chain, the following parameters were observed: an open circuit voltage (*V*_OC_) of 0.68 V, a *J*_SC_ of 8.79 mA cm^−2^, a fill factor (FF) of 37.60%, and a PCE of 2.36%. Devices incorporating IDC6CP-IC exhibited a higher *V*_OC_ of 0.77 V, a *J*_SC_ of 8.15 mA cm^−2^, and an FF of 46.10%, and achieved the highest PCE of 3.10%. These results underscore the potential of side-chain engineering to tune the aggregation behavior between URAs, thereby enhancing the photovoltaic efficiency of the OSCs. The EQE spectra for IDC6CP-IC demonstrates a broader photoresponse range of 400–850 nm compared to IDC8CP-IC. Additionally, the maximum EQE photoresponse of IDC6CP-IC is consistently higher than that of IDC8CP-IC across the photoresponse range, with respective enhancements of 20% and 16% (Figure 6a).

To explore the exciton dissociation process and charge complexation, the correlation between saturated photocurrent density (*J*_ph_) and effective voltage (*V*_eff_) for two devices was investigated, as shown in Figure 6c,d. The saturated photocurrent density, *J*_ph_, is defined by the equation *J*_ph_ = *J*_L_ − *J*_D_, where *J*_L_ and *J*_D_ represent the photocurrent density under illumination and dark conditions, respectively. The effective voltage, *V*_eff_, is given by *V*_eff_ = *V*_0_ − *V*_bias_, where *V*_0_ is the voltage at which *J*_ph_ is zero, and *V*_bias_ is the externally applied voltage bias. The dissociation probability, P_diss_, is calculated by dividing *J*_ph_ by the saturated photocurrent density (*J*_sat_) under short-circuit conditions. The P_diss_ values for devices based on IDC6CP-IC and IDC8CP-IC are 94.19% and 74.14%, respectively, indicating a higher exciton dissociation efficiency in the IDC6CP-IC device. Additionally, the charge complexation mechanism within the device was examined by assessing the dependence of short-circuit current density (*J*_SC_) on light intensity (P_light_) (Figure 6e). This relationship is typically represented by *J*_SC_∝P_lightα_, where α is a factor indicative of bimolecular recombination. When α approaches 1, it suggests minimal bimolecular recombination. As depicted in Figure 6f, the α values for devices based on IDC6CP-IC and IDC8CP-IC are 96.3% and 95.1%, respectively, demonstrating that both device types exhibit low bimolecular recombination. Under open-circuit conditions, the complexation index is described by the slope of the relationship *V*_OC_∝*β*K_B_T/(qlnPlight), where K_B_ is Boltzmann’s constant, T is the absolute temperature, and q is the elementary charge. Generally, a slope of 1 kT/q suggests dominant bimolecular recombination, whereas slopes approaching 2 kT/q indicate trap-assisted or unimolecular composites within the heterojunction. The slopes for the BHJs based on IDC6CP-IC and IDC8CP-IC are 1.734 kT/q and 1.244 kT/q, respectively. Devices using IDC8CP-IC demonstrate smaller slopes, indicative of minimal trap-assisted recombination and leading to a higher *J*_SC_. These findings reveal that while IDC6CP-IC devices have the most efficient exciton dissociation, their slightly lower *J*_SC_ compared to IDC8CP-IC devices is likely due to higher levels of trap-assisted recombination.

To assess the impact of alkyl chain length on charge transfer, electron mobility (*μ*_e_) and hole mobility (*μ*_h_) in the best-performing devices were measured using the space charge limiting current (SCLC) method (Table 2 and Figure S1). Charge transport behavior in the active layer is influenced by the respective donor and acceptor materials; therefore, comparing acceptors with identical backbones but different alkyl chains is valid as both devices utilize PBDB-T as the donor. Generally, balanced electron and hole mobilities enhance charge transport, contributing to a higher FF and *J*_SC_, and closer values of *μ*_e_ and *μ*_h_ which indicate a more balanced charge mobility across the device. For BHJs based on IDC8CP-IC and IDC6CP-IC, the measured hole mobilities are 1.22 × 10^−5^ and 1.62 × 10^−5^ cm^2^/V s, respectively, while the electron mobilities are 2.88 × 10^−5^ and 3.48 × 10^−5^ cm^2^/V s. These values demonstrate that both electron and hole transport in IDC6CP-IC exceed those in IDC8CP-IC, indicating superior charge transport capabilities. Furthermore, the ratios of *μ*_h_/*μ*_e_ for IDC8CP-IC and IDC6CP-IC-based BHJs are 2.36 and 2.14, respectively, suggesting that IDC6CP-IC exhibits more balanced and effective charge transport. This balance reduces interfacial space charge accumulation and minimizes carrier recombination, which can be attributed to stronger intermolecular aggregation facilitated by IDC6CP-IC. Additionally, the presence of long alkyl chains may attenuate intermolecular interactions, thereby complicating charge transport.

## 3. Conclusions

In conclusion, two A−DA’D−A type URAs, IDC8CP-IC and IDC6CP-IC, featuring differing side chains (2-hexyldecyl and 2-octyldodecyl), have been synthesized utilizing IID as the A’ unit, CPDT as the D unit, and DFIC as the terminal groups via DACH reactions, the Vilsmeier–Haack (V–H) reaction, and a Knoevenagel condensation. Both URAs exhibit suitable molecular planarity conducive to the formation of a 3D interpenetrating network, enhancing charge mobility. Specifically, the IDC6CP-IC film demonstrates a maximum absorption wavelength reaching 789.5 nm in the near-infrared spectrum with a pronounced shoulder peak, indicative of stronger molecular interactions and aggregation. Consequently, the URA with the 2-hexyldecyl side chain (IDC6CP-IC) achieved a higher PCE of 3.10%, in conjunction with PBDB-T, attributed to its superior external quantum efficiency, exciton dissociation efficiency, and balanced charge transfer, which collectively enhance the *V*_OC_ and FF. However, the *J*_SC_ of IDC6CP-IC was marginally lower due to increased trap-assisted recombination. These findings highlight the potential of side chain modifications in the A’ framework of A−DA’D−A type URAs to influence intermolecular stacking and achieve NIR absorption. This capability presents a distinct advantage over FRAs, underscoring the potential of IID-based URAs in the development of semi-transparent OSCs.

## Figures and Tables

**Table 1:** Optical and electrochemical properties of IDC8CP-IC and IDC6CP-IC.

Acceptors	λ_maxs_ (nm)	λ_maxf_ (nm)	λ_onsetf_ (nm)	ε (M^−1^ cm^−1^)	E_g_^opt^ (eV)	HOMO (eV)	LUMO (eV)
IDC8CP-IC	720.5	700.0, 761.5	900.0	6.16 × 10^5^	1.38	−5.43	−4.05
IDC6CP-IC	721.5	720.0, 789.5	930.0	6.15 × 10^5^	1.33	−5.42	−4.09

**Table 2:** Photovoltaic parameters of the optimized BHJs based on PBDB-T: IDC8CP-IC and PBDB-T: IDC6CP-IC.

BHJs	*V*_OC_ (V)	*J*_SC_ (mA cm^−2^)	FF (%)	PCE (%)	*μ*_h_ (×10^−5^ cm^2^V^−1^s^−1^)	*μ*_e_ (×10^−5^ cm^2^V^−1^s^−1^)
PBDB-T:IDC8CP-IC	0.68	8.79	37.60	2.36	1.22 ± 0.01	2.88 ± 0.01
PBDB-T:IDC6CP-IC	0.77	8.15	46.10	3.10	1.62 ± 0.01	3.48 ± 0.02

## Data Availability

The data presented in this study are available on request from the corresponding author.

## Supplementary Materials

The following supporting information can be downloaded at: https://www.mdpi.com/article/10.3390/molecules30020344/s1. Scheme S1: Synthetic route of IDC8CP-IC. Scheme S2: Synthetic route of IDC6CP-IC. Figure S1: (a) *J*^0.5^−*V* curves of the electron-only devices based on the two optimal blended films, and (b) *J*^0.5^−*V* curves of the hole-only devices based on the two optimal blended film. Figure S2: ^1^H NMR spectra of compound 3 in CDCl_3_. Figure S3: ^1^H NMR spectra of IDC8CP-IC in CDCl_3_. Figure S4: ^1^H NMR spectra of compound 5 in CDCl_3_. Figure S5: ^1^H NMR spectra of IDC6CP-IC in CDCl_3_. Figure S6: MALDI-TOF MS of IDC8CP-IC calcd. 2105.052, found 2106.191. Figure S7: MALDI-TOF MS of IDC6CP-IC calcd. 1992.836, found 1992.063. Figure S8: CV curves of ferrocene.
