# Supplementary material for: Achieving a Near-Infrared Absorption by A−DA’D−A Type Isoindigo-Based Small Molecular Acceptors for Organic Photovoltaics
Source: Molecules. 2025 Jan 16;30(2):344. doi: 10.3390/molecules30020344 (PMC11767318; doi:10.3390/molecules30020344)
Supplement: Supplementary file 1 [file molecules-30-00344-s001.zip › molecules-3392278-supplementary.pdf]

Materials and Instruments.

Unless otherwise specified, all conventional chemicals were purchased from Energy Chemical (Shanghai). The starting CPDT and IID were purchased from Derthon Technology Co Ltd (Shenzhen). Anhydrous toluene was obtained from treating conventional ones with CaH_2. The ^{1}H NMR and ^{13}C NMR spectra were recorded in Bruker Advance NMR spectrometer at 400 MHz frequency and obtained in chloroform-*d*. MS was performed on a Bruker Auto flex II using 2,5 dihydroxy benzoic acid or α cyano 4 hydroxycinnamic acid as the matrixes. Samples for MS were prepared by diluting the molecules in $CHCl_3$. Elemental analysis was taken on a Vario MICRO cube spectrophotometer. UV-vis absorption spectra were taken on a Shimadzu UV 2450 spectrophotometer. Theoretical calculations based on DFT methods have been performed for the compounds with Gaussian 09 program. Becke's three-parameter gradient corrected functional (B3LYP) with the 6-31G (d,p) basis set was used for geometric optimization. Cyclic voltammetry (CV) was measured on a CHI600A electrochemical workstation with Glassy carbon electrode, Pt wire, and Ag/Ag^+ electrode as working electrode, counter electrode, and reference electrode, respectively. The CV curves were recorded, which was calibrated by the ferrocene-ferrocenium (Fc/Fc^+) redox couple (4.8 eV below the vacuum level).

The current density-voltage (*J-V*) curves of photovoltaic devices were measured in the glovebox with Keithley 2400, under AM 1.5G illumination at 100 mW cm^{-2} irradiation using an Enli SS-F5-3A solar simulator, and the light intensity was calibrated with a standard Si solar cell with KG5 filter (made by Enli Technology Co., Ltd., Taiwan, and calibrated report can be traced to NREL). The external quantum efficiency (EQE) spectrum was measured using a QE-R Solar Cell Spectral Response Measurement System (Enli Technology Co., Ltd., Taiwan).

Device fabrication and characterization

Organic solar cells were fabricated on glass substrates commercially pre-coated with a layer of indium tin oxide (ITO), constructing the conventional structures of ITO/poly(3,4ethylenedioxy thiophene):poly(styrenesulfonate) (PEDOT:PSS)/BHJ/PDINO/Ag. Before fabrication, The ITO/glass substrates were ultrasonically cleaned using detergent, deionized water, acetone, and ethanol in that order for 30 minutes, then dried in an oven, and then treated in UV/ozone for 15 minutes. PEDOT:PSS precursor solution was rotationally coated on a clean and dry ITO substrate at 3500 rpm for 40 seconds and heated in air at 135°C for 10 minutes. The substrates were then transferred to a glove box. PBDB-T: acceptor (1:1.2 w/w) was prepared in 20 mg/mL chlorobenzene solution by stirring on a hot plate at 40 °C for at least 8 h. Then 0.5 vol.% 1-chlorobenzene (CN) was added to the chlorobenzene solution. The active layer was spin-coated at 3000 rpm for 60 s and then annealed at 100 °C for 10 min. PDINO was spin-coated at 3000 rpm for 60 s. Finally, Ag electrodes with a thickness of 100 nm were deposited by appropriate shadow masks to form a device

area of 0.043 cm2. The carrier mobility of the PBDB-T:receptor membrane was determined using the space charge limiting current (SCLC) method. The purely electronic devices were prepared in ITO/ZnO /PBDB-T:acceptor/PDINO/Ag structure and the hole devices were prepared in ITO/PEDOT:PSS/PBDB-T:acceptor/MoO_3/Ag structure. The device characteristics were extracted by modeling the dark current under forwarding bias using the SCLC expression described by the Mott-Gurney law:

$$J = \frac{9}{8}\varepsilon_0\varepsilon_r\mu_0\frac{V^2}{L^3}$$

where ε_r is the average dielectric constant of the blended film, ε_0 is the dielectric constant of free space (8.85×10^{-12} F m^{-1}), μ_o is the hole or electron mobility, L is the film thickness of the active layer, and V is the applied voltage.

Experimental Section

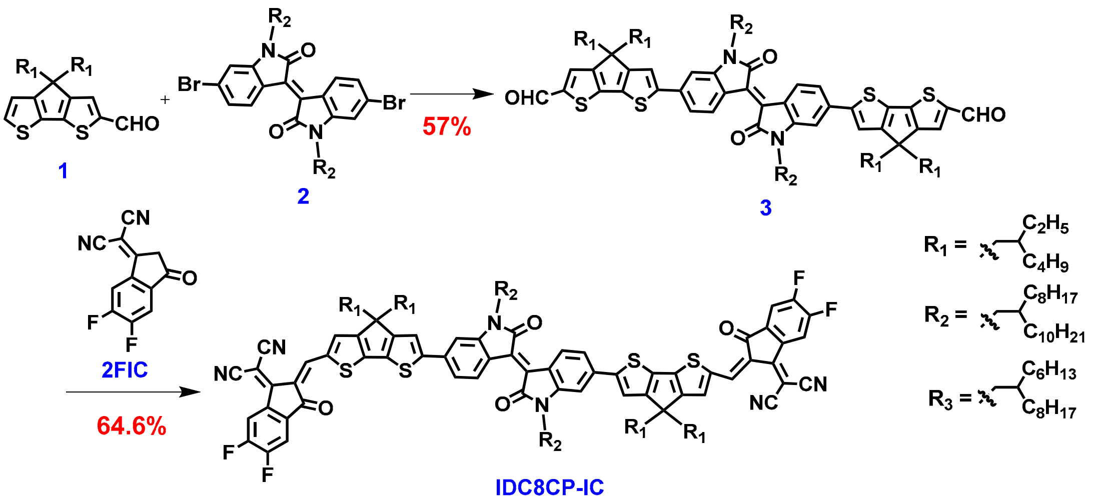

Scheme S1. Synthetic route of **IDC8CP-IC**.

Synthesis of compound 1

To a cold solution of cyclopentadithiophene (337.97 mg, 0.94 mmol) and N,N-dimethylformide (82.40 mg, 1.13 mmol) in 1,2-dichloroethane (5.7 mL) at 0 °C was added phosphorus chloride oxide (0.19 mL) under argon. The reaction solution was stirred at the same temperature for 4 h and then saturated potassium acetate aqueous solution (5.7 mL) added. The mixture was further stirred at room temperature for 2 h. The crude product was extracted into dichloromethane, and the organic layer was washed with brine and water, and dried over anhydrous sodium sulfate. After removing solvent under reduced pressure, the residue was purified by column chromatography (petroleum ether (PE): dichloromethane (DCM), 2/3, v/v) on silica gel to yield a colorless oil (219.92 mg, 54.4% yield). ^{1}H NMR (400 MHz, $CDCl_3$) δ 9.82 (s, 1H), 7.56 (t, J = 3.7 Hz, 1H), 7.37 (d, J = 4.9 Hz, 1H), 6.99 (dt, J = 4.9, 2.5 Hz, 1H), 1.91 (dt, J = 9.4, 3.4 Hz, 4H), 1.09-

0.77 (m, 18H), 0.75-0.56 (m, 12H).

Synthesis of compound 3

Compound **1** (76.2 mg, 0.177 mmol), Compound **2** (79.47 mg, 0.081 mmol), trimethylacetic acid (PivOH) (8.25 mg, 0.081 mmol), Cs_2CO_3 (210.95 mg, 0.648 mmol), $Pd_2(dba)_3$ (7.49 mg, 0.0081 mmol), P(*o*-$CH_3OPh)_3$ (5.66 mg, 0.0162 mmol), and dry toluene (6 mL) were all added into a 25 mL two-necked round-bottomed flask, which were heated to 120 °C under a nitrogen atmosphere for 24 hours. Then the reaction mixture was cooled down to room temperature, poured into water, extracted with DCM, washed with brine, and dried over Na_2SO_4. After removal of the organic solvent, the residue was purified by silica gel column chromatography with PE: DCM (2:3, v/v) as the eluent to give an orange red liquid (77.6 mg, 57.0% yield). ^{1}H NMR (400 MHz, $CDCl_3$) δ 9.86 (s, 2H), 9.21 (d, J = 8.4 Hz, 2H), 7.59 (s, 3H), 7.32 (s, 3H), 6.98 (s, 2H), 3.74 (d, J = 7.1 Hz, 4H), 2.01-1.85 (m, 12H), 1.50-1.02 (m, 96H), 0.85-0.57 (m, 36H).

Synthesis of IDC8CP-IC

The Compound **3** (77.6 mg, 0.046 mmol) and 2FIC units (22.33 mg, 0.097 mmol) were dissolved in dry toluene (3 mL). $BF_3{\cdot}OEt_2$ (65.60 mg, 5 equiv of the IC units) and acetic anhydride (0.046 mL) were added, and the reaction mixture was stirred at room temperature for 15 min. Then, the reaction mixture was added dropwise into methanol with stirring. The precipitate was collected, after removal of the organic solvent, the residue was purified by silica gel column chromatography with PE: DCM (2:1, v/v) as the eluent to give a blue-black solid (62.8 mg, 64.6% yield).

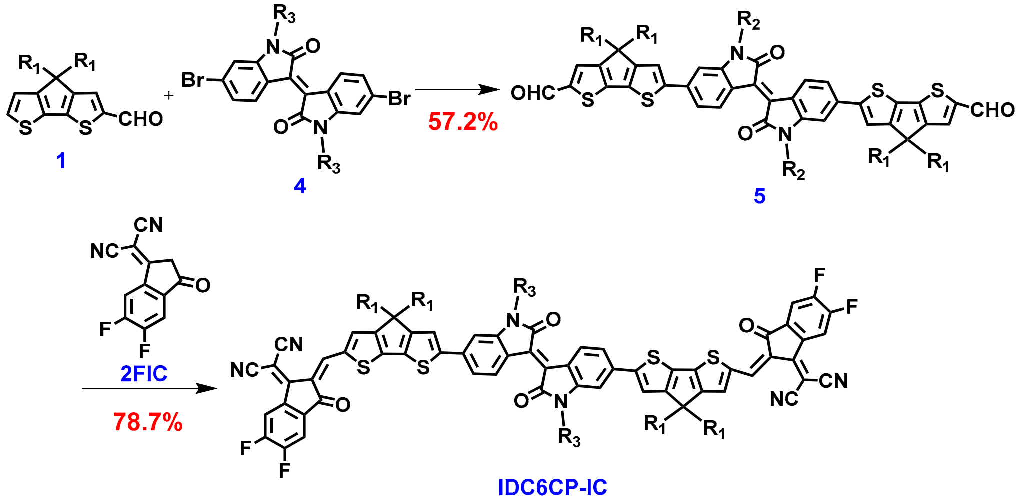

Scheme S2. Synthetic route of **IDC6CP-IC**.

Synthesis of compound 5

The synthetic route of Compound **5** is similar to Compound **3**. Compound **1** (125.3 mg, 0.291 mmol), Compound **4** (115.71 mg, 0.133 mmol), PivOH (13.55 mg, 0.133 mmol), Cs_2CO_3 (346.37 mg, 1.065 mmol), $Pd_2(dba)_3$ (12.31 mg, 0.0133 mmol), P(*o*-$CH_3OPh)_3$ (9.31 mg, 0.027 mmol), and dry toluene (8 mL) were all added into a 25 mL two-necked round-bottomed flask, which were

heated to 120 °C under a nitrogen atmosphere for 24 hours. Then the reaction mixture was cooled down to room temperature, poured into water, extracted with DCM, washed with brine, and dried over Na_2SO_4. After removal of the organic solvent, the residue was purified by silica gel column chromatography with PE: DCM (2:3, v/v) as the eluent to give an orange red liquid (119.4 mg, 57.2% yield). ^{1}H NMR (400 MHz, $CDCl_3$) δ 9.84 (d, J = 10.5 Hz, 2H), 9.21 (d, J = 8.4 Hz, 2H), 7.59 (t, J = 3.6 Hz, 3H), 7.32 (s, 3H), 6.98 (s, 2H), 3.74 (d, J = 7.1 Hz, 4H), 2.06-1.91 (m, 12H), 1.39-1.20 (m, 80H), 0.85-0.57 (m, 36H).

Synthesis of IDC6CP-IC

The Compound **5** (119.4 mg, 0.076 mmol) and 2FIC units (36.95 mg, 0.161 mmol) were dissolved in dry toluene (5 mL). $BF_3{\cdot}OEt_2$ (108.88 mg, 5 equiv of the IC units) and acetic anhydride (0.076 mL) were added, and the reaction mixture was stirred at room temperature for 15 min. Then, the reaction mixture was added dropwise into methanol with stirring. The precipitate was collected, after removal of the organic solvent, the residue was purified by silica gel column chromatography with PE: DCM (1:1, v/v) as the eluent to give a blue-black solid (119.4 mg, 78.7% yield). ^{1}H NMR (400 MHz, $CDCl_3$) δ 9.22 (d, J = 8.4 Hz, 2H), 8.89 (s, 2H), 8.51 (dd, J = 10.0, 6.5 Hz, 2H), 7.66 (t, J = 7.5 Hz, 4H), 7.40-7.31 (m, 4H), 7.00 (s, 2H), 3.76 (d, J = 6.9 Hz, 4H), 2.02 (s, 14H), 1.40-0.95 (m, 81H), 0.87-0.64 (m, 36H). MALDI-TOF MS: $[M]^+$ calcd for $C_{124}H_{150}F_4N_6O_4S_4$, 1992.84, found m/z 1992.06.

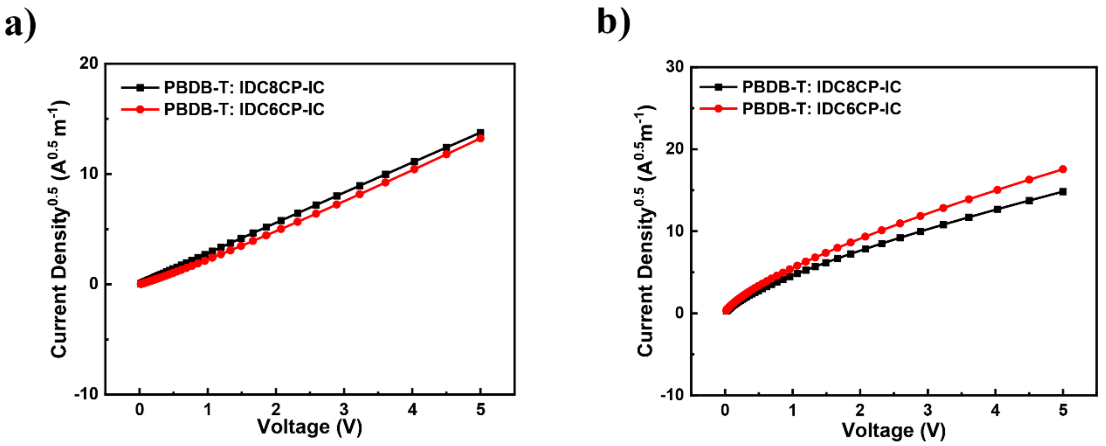

Figure S1 (a) $J^{0.5}$–V curves of the electron-only devices based on the two optimal blended films. (b) $J^{0.5}$–V curves of the hole-only devices based on the two optimal blended films.

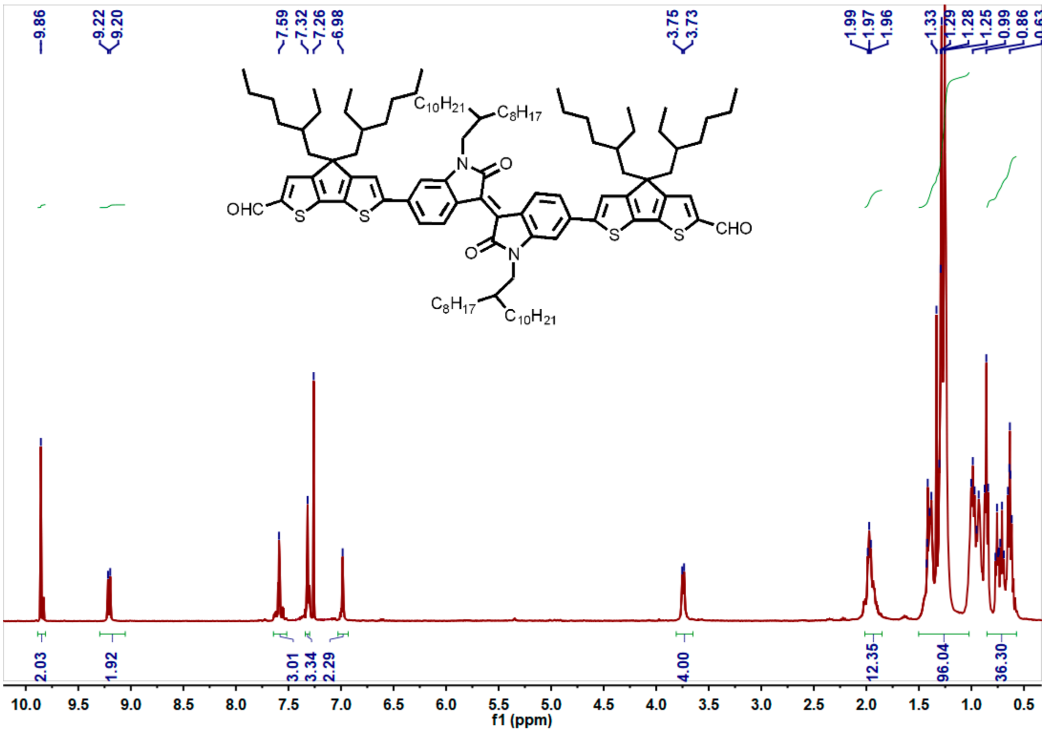

Figure S2 ^{1}H NMR spectra of compound **3** in $CDCl_3$.

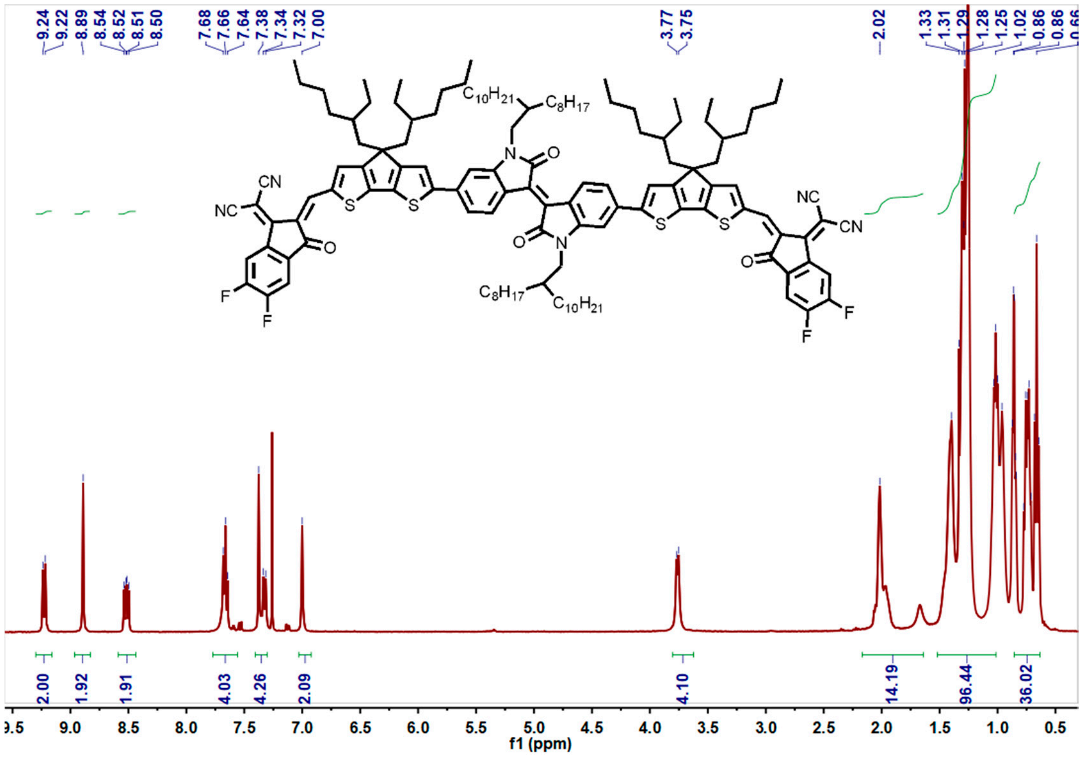

Figure S3 ^{1}H NMR spectra of **IDC8CP-IC** in $CDCl_3$.

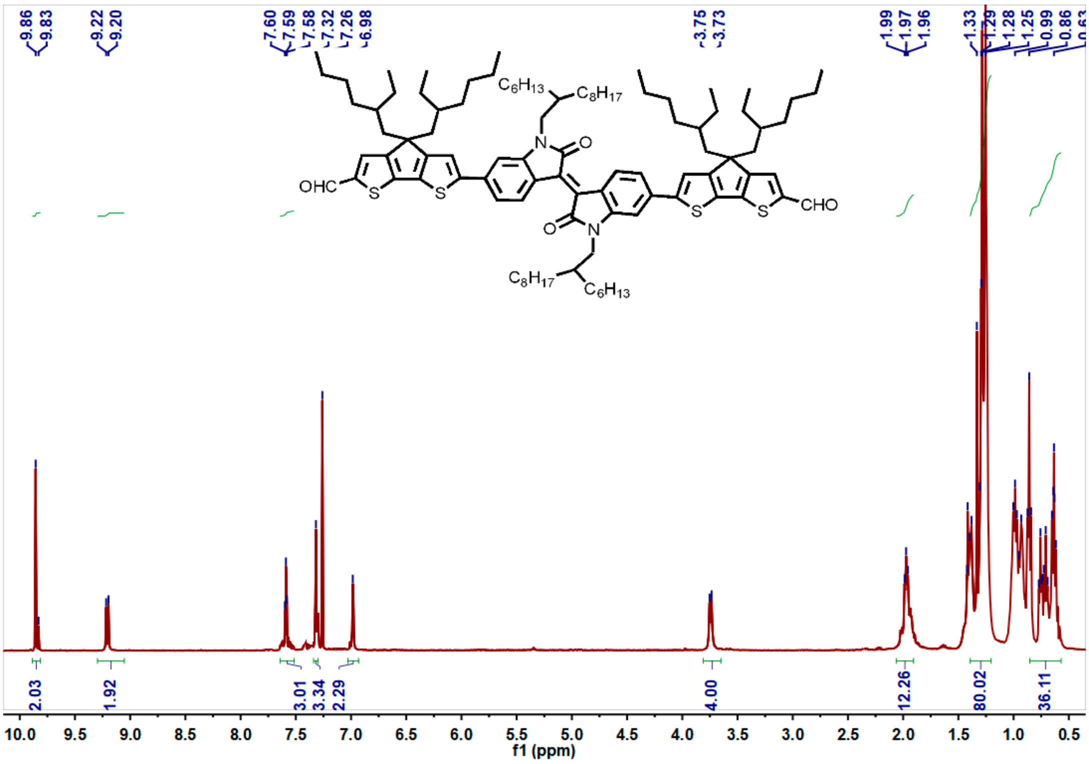

Figure S4 ^{1}H NMR spectra of compound **5** in $CDCl_3$.

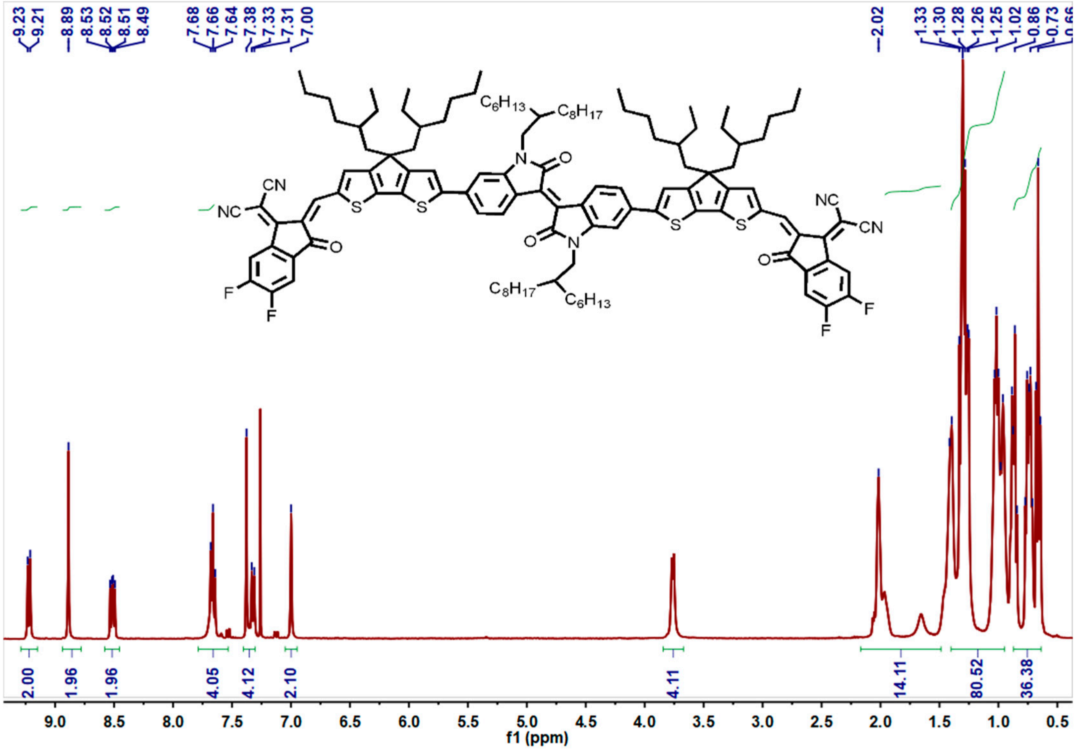

Figure S5 ^{1}H NMR spectra of **IDC6CP-IC** in $CDCl_3$.

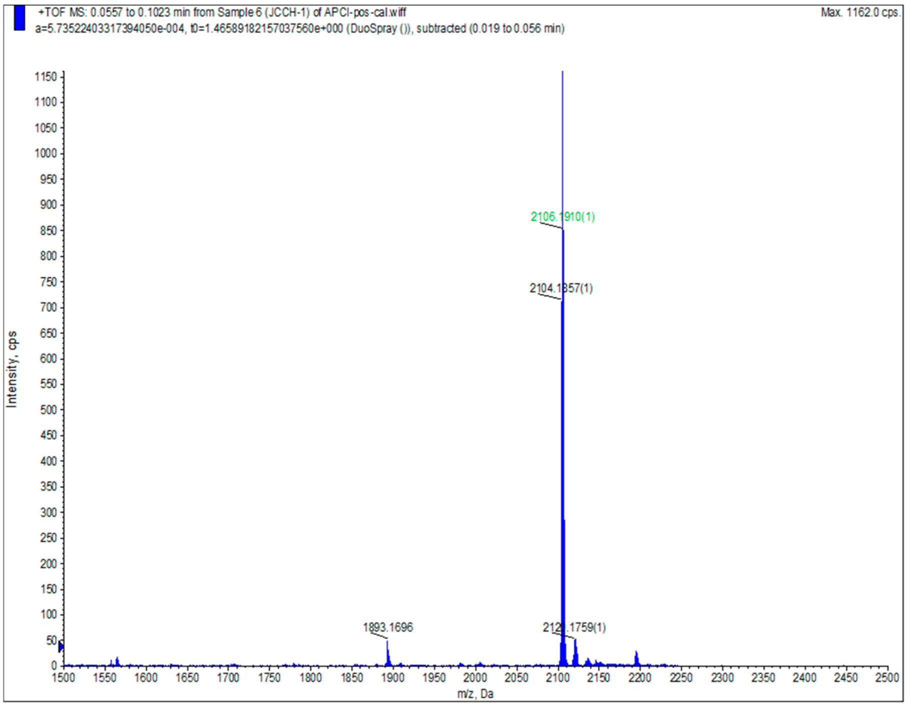

Figure S6 MALDI-TOF MS of **IDC8CP-IC** calcd. 2105.052, found 2106.191.

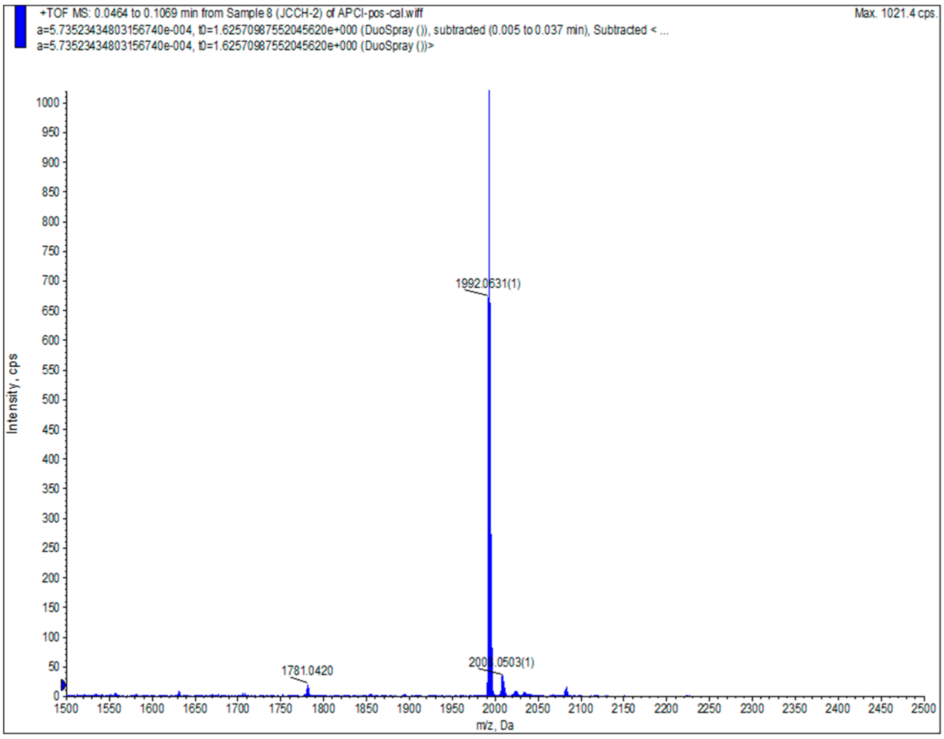

Figure S7 MALDI-TOF MS of **IDC6CP-IC** calcd. 1992.836, found 1992.063.

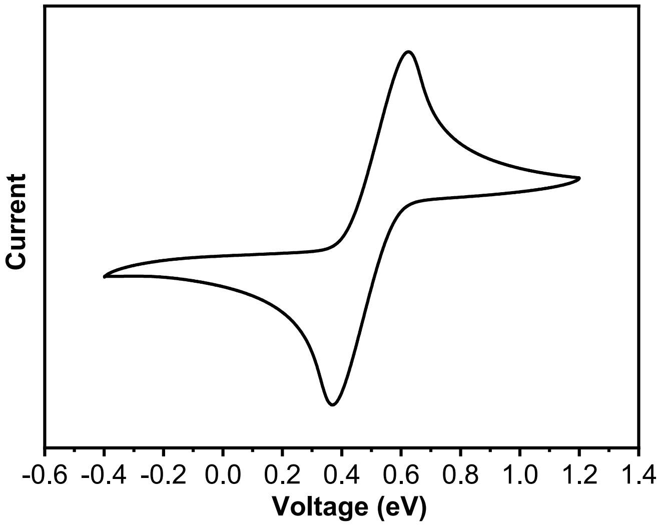

Figure S8 CV curves of ferrocene
